# Using cfRNA as a tool to evaluate clinical treatment outcomes in patients with metastatic lung cancers and other tumors

**DOI:** 10.20517/cdr.2021.78

**Published:** 2021-12-13

**Authors:** Luis E. Raez, Kathleen Danenberg, Daniel Sumarriva, Joshua Usher, Jacob Sands, Aurelio Castrellon, Pablo Ferraro, Adriana Milillo, Eric Huang, Patrick Soon-Shiong, Sandeep Reddy, Peter Danenberg

**Affiliations:** ^1^Thoracic Oncology Program, Memorial Cancer Institute/Memorial Healthcare System, Florida International University, Miami, FL 33199, USA.; ^2^Nant Health, Culver City, 2040 E Mariposa Ave, El Segundo, CA 90245, USA.; ^3^Thoracic Oncology Program, Dana Farber Cancer Institute, Harvard Medical School, Boston, MA 02215, USA.; ^4^Burning Rock, Beijing 100022, China.; ^5^Department of Biochemistry and Molecular Medicine, University of Southern California, Los Angeles, CA 90089, USA.

**Keywords:** cfRNA, cfDNA, liquid biopsies, NSCLC, PD-L1

## Abstract

**Aim: **We report an exploratory analysis of cfRNA as a biomarker to monitor clinical responses in non-small cell lung cancer (NSCLC), breast cancer, and colorectal cancer (CRC). An analysis of cfRNA as a method for measuring PD-L1 expression with comparison to clinical responses was also performed in the NSCLC cohort.

**Methods: **Blood samples were collected from 127 patients with metastatic disease that were undergoing therapy, 52 with NSCLC, 50 with breast cancer, and 25 with CRC. cfRNA was purified from fractionated plasma, and following reverse transcription (RT), total cfRNA and gene expression of PD-L1were analyzed by real-time polymerase chain reaction (qPCR) using beta-actin expression as a surrogate for relative amounts of cfDNA and cfRNA. For the concordance study of liquid biopsies and tissue biopsies, the isolated RNA was analyzed by RNAseq for the expressions of 13 genes. We had to close the study early due to a lack of follow-up during the Covid-19 pandemic.

**Results: **We collected a total of 373 blood samples. Mean cfRNA PCR signals after RT were about 50-fold higher than those of cfDNA. cfRNA was detected in all patients, while cfDNA was detected in 88% of them. A high concordance was found for the expression levels of 13 genes between blood and solid tumor tissue. Changes in cfRNA levels followed over the course of treatments were associated with response to therapy, increasing in progressive disease (PD) and falling when a partial response (PR) occurred. The expression of PD-L1 over time in patients treated with immunotherapy decreased with PR but increased with PD. Pre-treatment levels of PD-L1 were predictive of response in patients treated with immunotherapy.

**Conclusion: **Changes in cfRNA correlate with clinical response to the therapy. Total cfRNA may be useful in predicting clinical outcomes. *PD-L1 *gene expression may provide a biomarker to predict response to PD-L1 inhibition.

## INTRODUCTION

Detection of oncogenic driver genomic alterations has historically been accomplished with tumor tissue-based tests, which are highly dependent on the amount of tumor tissue recovered in the biopsy after the tissue was first analyzed to establish the diagnosis^[1]^. This can be challenging in cases with limited tissue availability. However, the analysis of genetic material in the blood provides less invasive testing as a collection method for “liquid biopsies”^[2]^. Circulating cell-free DNA (cfDNA) and cfRNA are the results of apoptotic and necrotic events, which are increased in cancer due to both a higher replication rate of the tumor cells and the release of tumor-derived material, including nucleic acids, into the blood. The combination of high sensitivity techniques for detection, such as digital droplet polymerase chain reaction (PCR) and next-generation sequencing (NGS) techniques, have led to increasing use of cfDNA to detect molecular alterations with clinical value^[3-5]^. In non-small cell lung cancer (NSCLC), cfDNA can be used to detect both oncogenic driver mutations and resistance mutations. The value of these strategies has been well validated for genes like EGFR and now is well studied for KRAS but not only for diagnostic purposes but also for evaluation of resistance like T790 mutations after the use of tyrosine kinase inhibitors^[6-10]^.

Early change in cfDNA volume has been shown to predict response to chemotherapy in patients with metastatic colorectal cancer (CRC)^[11,12]^. However, sometimes it is difficult to isolate enough cfDNA from the blood for analysis. There are several potential advantages in the analysis of cfRNA compared with cfDNA. First, cfRNA may be present at higher levels than the corresponding gene in cfDNA. cfRNA should contain only those mutations that are consequential for tumor development. Importantly, the quantitative analysis of genes in RNA allows the measurement of tumor gene expressions, some of which are often elevated in tumors compared to normal tissues. The presence of cfRNA may increase the yield of gene expression information, including cases where the amount of cfDNA is insufficient for detection.

PD-L1 is a clinically meaningful biomarker that has treatment implications for solid tumors, including NSCLC, but analysis currently requires tissue availability for immunohistochemical (IHC) staining. An effective blood-based test that can categorize PD-L1 level of expression would reduce or eliminate the need for tumor tissue biopsies when determining treatment options. As an immune suppression mechanism, the expression of PD-L1 is elevated in many types of cancer and is often correlated with poor prognosis, and is predictive of responses to the antibodies against PD-1/PD-L1^[13,14]^. Therapies that block this interaction have demonstrated very promising clinical activity in several cancer types^[15,16]^. Preliminary data from our group on the measurement of PD-L1 by reverse transcription (RT)-PCR using cfRNA from 760 patients demonstrated PD-L1 expression by cfRNA was similar to levels detected by IHC analysis of tumor tissue^[17]^. Gene expression in plasma has also demonstrated a correlation with response to nivolumab similar to IHC measurement of PD-L1 in tumor tissue^[18]^. These exploratory results paved the way for further studies aimed at determining whether monitoring the quantitative levels of PD-L1 mRNA in the blood before and during treatment can provide additional predictive value for anti-PD-L1 therapy^[18]^. Moreover, the lack of measurable PD-L1 cfRNA in cancer-free individuals suggests that the presence of PD-L1 cfRNA in the blood may be a potential marker for detecting the recurrence of cancer since we have not detected PD-L1 in cfRNA from healthy individuals^[17]^. In the current study, we compared levels of cfDNA and cfRNA to assess the utility of cfRNA as a biomarker to follow therapy outcomes in NSCLC, breast cancer, and CRC. We also measured *PD-L1 *gene expression in cfRNA to evaluate its relationship to immunotherapy outcomes.

## METHODS

### Blood sample processing

Blood samples (20 cc) from patients with metastatic NSCLC, breast cancer, and CRC were drawn into RNA and DNA BCT tubes (Streck) pre-treatment and at three-month intervals or progression for a year while patients were monitored for their clinical therapy outcomes by CT scan as a standard of care for these solid tumors. The two tubes contained a proprietary nucleic acid preservation cocktail and were transferred to Liquid Genomics, Inc. as soon as possible (usually the first 24 h after drawing the sample). After blind accessioning, all samples proceeded to the isolation process within five days after collection. A two-dimensional bar code was placed on all plasma samples for automatic identification. Streck BCT tubes with the whole blood were centrifuged to fractionate plasma at 16,000 rcf for 20 min. cfRNA was extracted from 2 cc of plasma with a proprietary in-house protocol developed by Liquid Genomics, Inc., specially designed to remove potential contaminating blood cells during the extraction. All nucleic acids were kept in bar-coded matrix storage tubes. RNA was immediately reverse-transcribed to complementary DNA (cDNA) upon extraction, and cDNA was stored at 4 °C. The remaining plasma was stored at -80 °C and yielded cfRNA with concordant gene expressions for over two years of storage.

### PCR quantitation

Quantitative real-time RT-PCR was used to detect gene expressions in cfRNA, as described previously^[17]^. Briefly, PCR analysis was accomplished using random primed reverse transcription and amplification with gene-specific primer-probes. We analyzed the relative cfRNA and cfDNA content of patients’ blood by comparing their respective PCR signals for the house-keeping gene beta-actin (Β-actin). The RNA and DNA were separately quantitated in plasma isolated from two identical blood samples taken from each patient. Changes in relative levels of cfRNA, cfDNA, and gene expressions quantitated by qPCR were correlated with patients’ response to therapy as per RECIST criteria: complete response (CR), partial response (PR), stable disease (SD), and disease progression (PD), as determined by CT scans. The current study was approved by the Memoria Health Care System Institutional Review Board. We were unable to collect several blood samples during the year of follow-up due to the COVID-19 pandemic in 2020 that limited in-person follow-up as most visits were done by telemedicine, preventing the opportunity to collect blood samples in some cases. The decision was made to stop enrollment and report all the available preliminary data in this manuscript. A total of 373 blood samples were collected, including 154 breast cancer samples, 135 NSCLC samples, and 84 CRC samples among 127 patients.

### RNAseq analysis

In a concordance study, whole transcriptome RNA seq analysis was performed on 87 tissue-plasma paired samples by Burning Rock Biotech LTD. Cell-free nucleic acids were extracted from plasma and treated with DNase to remove cfDNA and genomic DNA. RNA was reverse-transcribed using random hexamer primers to capture the whole transcriptome for each sample. The resulting cDNA was converted into DNA libraries and amplified. Depletion of ribosomal and mitochondrial RNA sequences was performed. This procedure was used to create and analyze RNAseq libraries from matched tumor tissue RNA. RNASeq analysis of the total transcriptome was performed in 87 tissue - plasma paired samples (including lung cancer and colon cancer). Representative biomarker expressions were compared in paired tissue and plasma samples.

## RESULTS

**Figure 1:**
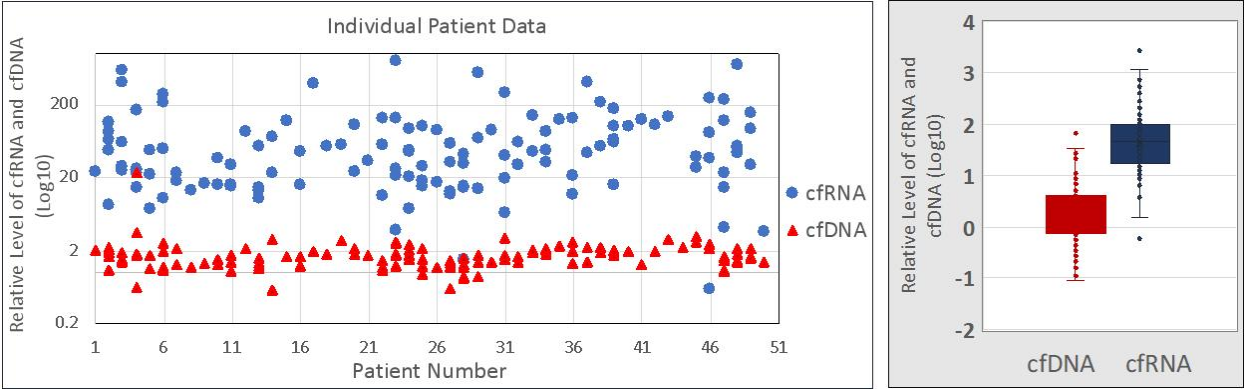
PCR signals of beta-actin from cfRNA (as its cDNA) and from cfDNA in identical aliquots of patients’ plasma. *P *< 0.001 for the difference in median signal values (Wilcoxon/Kruskal-Wallis rank sums). PCR: Polymerase chain reaction.

**Figure 2:**
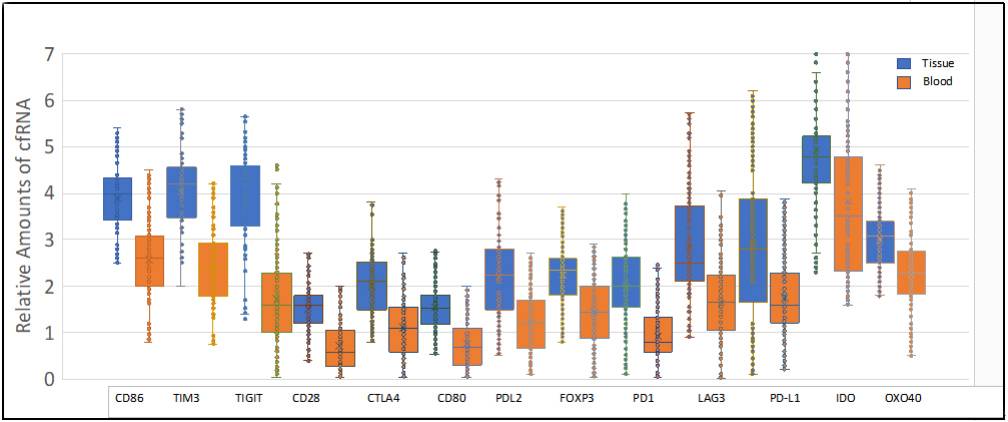
Concordance between the expressions of 13 genes in paired plasma and tissue samples a is determined by RNAseq.

**Figure 3:**
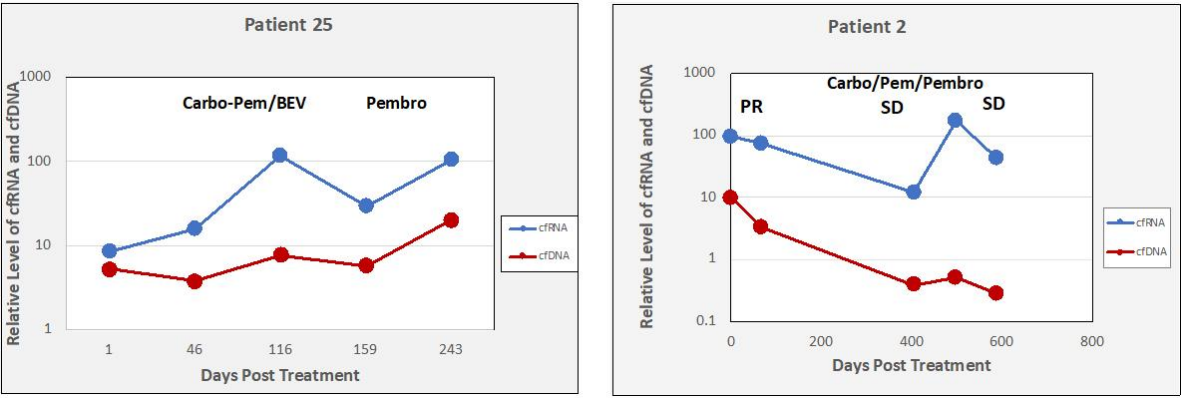
Changes in levels of cfRNA and cfDNA during therapy. cfRNA and cfDNA were measured at initiation of therapy and at various times during the chemotherapy using PCR amplification of B-actin. Treatment efficacy was determined by CT scans. PCR: Polymerase chain reaction.

**Figure 4:**
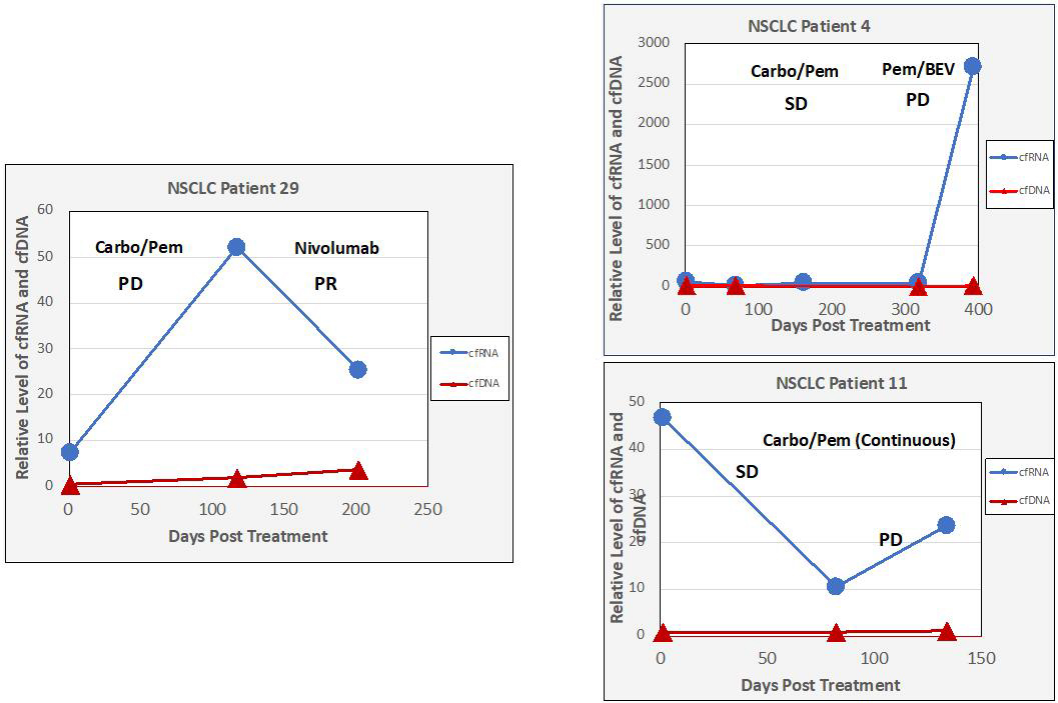
Changes in cfRNA levels during therapy when cfDNA is unmeasurable. cfRNA and cfDNA were measured at initiation of treatment and at various times during chemotherapy using PCR amplification of Β-actin. Treatment efficacy of chemotherapy regimens was determined by CT scans. PCR: Polymerase chain reaction.

A total of 127 patients with metastatic disease that were undergoing active therapy were enrolled in the study: 52 with NSCLC, 50 with breast cancer, and 25 with CRC [Table 1]. Most of the patients were female (63%) and non-Hispanic (67%). Among individuals with NSCLC, most of them (87%) had prior or current smoking histories. The median cfRNA signal in blood was about 50-fold higher than the cfDNA median (*P *< 0.001) [Figure 1]. Most of the cfRNA and cfDNA values fall within about a 10-fold range, with a few high and low outliers. cfRNA was quantifiable in all samples, while cfDNA was too low to be quantifiable in 12% of the samples. In 10 healthy volunteers, cfRNA was too low to be accurately measured (ct > 36). A concordance study done by our group between biomarker expression in blood and tumor tissue using RNAseq analysis of the total transcriptome in 87 tissue - plasma paired samples (including NSCLC and CRC) shows 13 genes, including PD-L1, PDL2, and PD [Figure 2]. The qualitative concordance between tissue and plasma expressions reached 84%, and the quantitative relative expression concordance reached 76% (Unpublished data). We had a total of 373 samples collected for the study before it was suspended. To evaluate the concordance between any variations in cfRNA and cfDNA content with the results of the therapy, cfRNA and cfDNA were measured at initiation of treatment and at various times during chemotherapy treatments using PCR amplification of Β-actin [Figures 3 and 4]. Treatment efficacy of the chemotherapy regimen as determined by CT scans was compared with changes in cfRNA and cfDNA levels. As an example, patient 2 [Figure 3] experienced an initial PR with a concomitant decline in both total cfRNA and cfDNA, followed by a parallel increase in both nucleic acids as the patient transitioned into SD status. Patient 25 suffered PD under the therapy with substantial increases in cfRNA and smaller increases in cfDNA during this time. These results show that changes in total cfRNA and cfDNA levels are concordant when cfDNA is measurable, and thus that cfRNA may be a valid monitor of the efficacy of treatment over time. Notably, the PCR signals of cfDNA were considerably lower than the cfRNA signals.

**Table 1:** Demographic information of the patients enrolled in the study

		**Lung cancer** ***n *** **= 52 (%)**	**Breast cancer** ***n *** **= 50 (%)**	**Colon cancer** ***n *** **= 25 (%)**	**Total** ***n *** **= 127**
Gender	Male	31 (60%)	3 (6%)	13 (52%)	47 (37%)
	Female	21 (40%)	47 (94%)	12 (48%)	80 (63%)
Tobacco	Smoker	7 (13%)	n/a	n/a	7 (13%)
	Former smoker	45 (87%)	n/a	n/a	45 (87%)
Ethnicity	Hispanics	13 (39%)	20 (40%)	9 (36%)	43 (33%)
	Non-Hispanics	25 (75%)	30 (60%)	16 (64%)	85 (67%)

**Figure 5:**
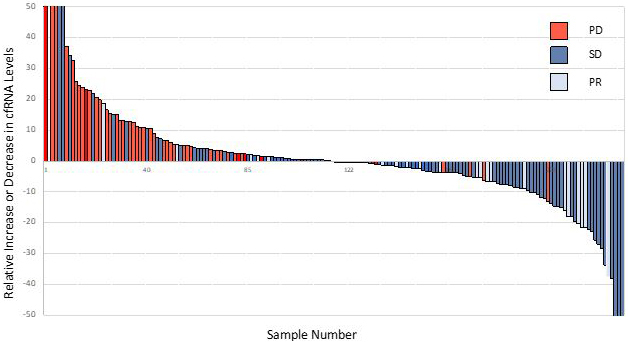
Changes in total cfRNA *vs*. outcome in response to various therapies across different tumor types. The bars represent analyses of 154 BC, 84 CRC, 135 NSCLC patient samples. BC: Breast cancer; CRC: colorectal cancer; NSCLC: non-small cell lung cancer.

**Figure 6:**
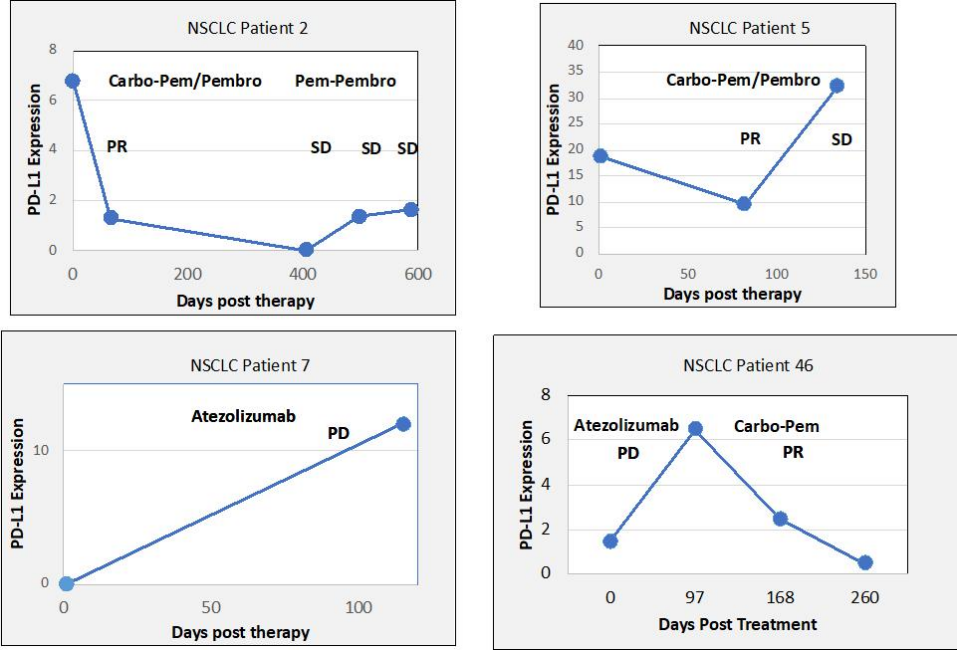
Association of response to chemotherapy with *PD-L1* gene expression determined using cfRNA in patients’ blood draws.

**Figure 7:**
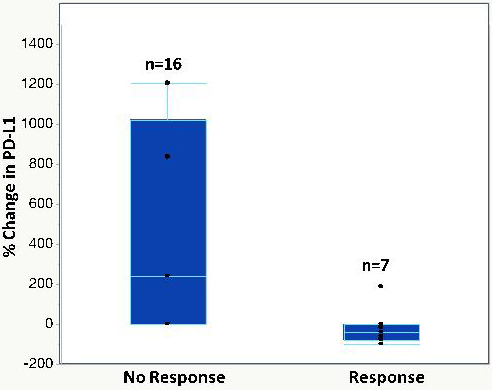
Measurement of *PD-L1* gene expressions in NSCLC patients during treatments with immunotherapy agents. Chemotherapy includes carbo-pemetrexed; targeted therapy includes erlotinib, osimertinib, and crizotinib. NSCLC: Non-small cell lung cancer.

**Figure 8:**
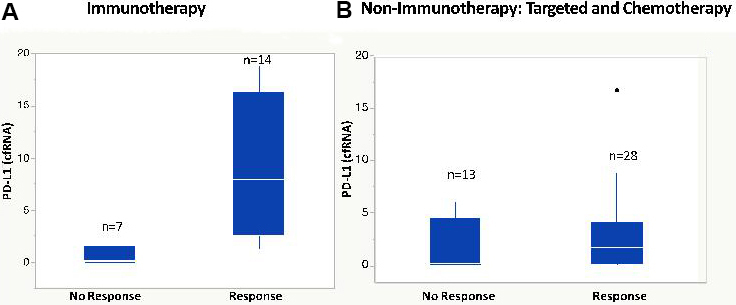
Pre-treatment measurement of *PD-L1* gene expressions. (A) NSCLC patients treated with immunotherapy. *P* = 0.003 for the difference in median values. (Wilcoxon /Kruskal Wallis Rank Sums). Immunotherapy includes pembrolizumab, nivolumab, atezolizumab. (B) Non-immunotherapy of NSCLC patients. NSCLC: Non-small cell lung cancer.

In 6/48 blood samples from individuals with NSCLC (12%), the PCR signal for cfDNA was out of the range of PCR quantitation, but cfRNA was still robustly quantifiable. In three patients without quantifiable cfDNA levels, changes in cfRNA were readily followed [Figure 4]. Patient 29 showed an increase in cfRNA while experiencing PD under carbo/pemetrexed but then a decrease in cfRNA when a switch to nivolumab induced a PR. In both patients 4 and 11, cfRNA levels began to increase with the onset of PD. These results indicate that cfRNA may be a useful biomarker for chemotherapy efficacy when cfDNA is too low to measure. Correlation between response to therapy and changes in cfRNA levels using data from 154 breast cancer, 84 CRC, and 135 NSCLC patient samples is noted [Figure 5]. An increase in cfRNA was predominantly associated with PD, while the cfRNA levels in the majority of PR or SD cases did not increase but spanned a spectrum of little or no change to a marked decrease (*P *< 0.001, Kruskal-Wallis test). From these data, the sensitivity and specificity of using changing levels of cRNA to predict the outcome of therapy are calculated to be 95.8% and 93.3%, respectively. One of the advantages of cfRNA analysis is that it can be used to measure the expression levels of specific genes. PD-L1 expression was monitored throughout a course of immunotherapy in 5 individuals with NSCLC [Figure 6]. In the three patients of this group who had a PR to the therapy, either at first (pts. 2 and 5) or following unsuccessful initial therapy (pt. 46), the occurrence of the PR was associated with a decrease in PD-L1 expression, while an increase in PD-L1 occurred in the two patients who had PD (pts. 7 and 46). Especially interesting in this regard is patient 46, whose initial PD was accompanied by a steep increase in PD-L1, followed by an equally steep decrease in PD-L1after a change of therapy resulted in a PR. The association between increasing PD-L1 expression and PD was confirmed in a larger set of patients treated with immunotherapies [Figure 7]. Pre-treatment levels of PD-L1 expression correlated with response to immunotherapies [Figure 8A]. High PD-L1 expression was associated with response, while low PD-L1 correlated with PD. PD-L1 expression was not predictive in patients not receiving immunotherapies [Figure 8B].

## DISCUSSION

In recent years, cfDNA has been widely studied as a source for determining cancer-related genomic alterations, including point mutations, copy number variations, and methylation markers^[19]^. However, there are a number of practical technical problems associated with the use of cfDNA, the main ones being that cfDNA is substantially fragmented (ca.160 bp), its concentrations are typically low (< 10 ng/mL of plasma), and the fraction of tumor-derived cfDNA in a typical patient’s blood draw is often quite small (often < 1%)^[20]^. These issues represent potential obstacles for application to early-stage cancer detection or analysis. The presence of cfRNA may serve as a more sensitive means of blood-based screening for cancer, but this will require much more extensive study. Another problem has been the ability to distinguish informative cfDNA mutations from a background of benign variants such as clonal hematopoiesis^[21]^. The detection of cfRNA may not have the same potential limitation, but further research and experience are necessary before drawing conclusions. DNA contains only one copy of a gene, or a few copies in the case of gene amplification, while expressed genes can be transcribed many times and moreover, overexpression of specific genes often occurs in tumors, leading to further amplification of tumor-derived RNA signals in the blood. This approach to cancer detection and characterization was developed in a recent study in which cfRNA biomarker genes were identified that were highly expressed in a tissue and subtype-specific manner in breast and lung cancer patients, allowing not only increased detection capability but also identification of the different cancers^[22,23]^.

Full-length mRNAs can be recovered from plasma, indicating that cfRNA is relatively stable in blood, possibly being protected by binding to proteins or by tightly wound secondary structures^[24,25]^. RNA storage tubes (Streck RNA BCT tubes) that stabilize the cfRNA and also prevent hemolysis provide further opportunity for stabilizing cfRNA for evaluation^[26]^. The results of this study are consistent with the expectation that cfRNA is often more abundant than cfDNA due to multiple transcription events. We found that the mean Β-actin signal from cfRNA was about 50 times greater than from the cfDNA [Figure 1], although it should be noted that this signal ratio does not indicate the actual relative amounts of cfRNA and cfDNA in plasma because of the extra amplification step when the RNA is reverse transcribed to its cDNA. Nevertheless, this difference in signal strength between cfRNA and cfDNA potentially results in greater sensitivity. For example, the higher signal strength of cfRNA may enhance the ability to detect and monitor post-surgical minimal residual disease (MRD), which demands highly sensitive detection of mutations to reliably predict disease relapse^[27]^. We observed cases in which cfRNA was quantifiable, and changes correlated with RECIST-defined responses, whereas cfDNA was not detectable [Figure 4], but the reliability of this requires more study. The concordance between biomarkers in cfRNA and tumor tissue is critical if cfRNA analysis is to be useful for cancer detection and monitoring. In this study, NGS analysis [Figure 2] showed a good concordance between a number of gene expressions in cfRNA and in the paired tissue samples. Our previous study on PD-L1 expression in cfRNA from various cancers also showed a good correlation between relative levels of *PD-L1 *gene expression in cfRNA and IHC analysis of PD-L1 in the corresponding tumor tissues^[18]^. Recently, Larson *et al*.^[22] ^identified tissue- and cancer-specific genes whose levels in plasma correlated with their RNA expression in matched tissue. These results, showing that the expression of genes in tumor tissue is also reflected in the cfRNA, further advocate the use of cfRNA for enhanced cancer detection in patients who have low amounts of cfDNA. To evaluate its usefulness as a treatment monitoring tool, we try to follow total cfRNA in individuals with cancer over the course of their treatment. There were multiple time points of consistency between cfRNA levels and RECIST responses that occurred regardless of the tumor type or treatment regimen. The increasing cfRNA signal associated with PD presumably reflects a rising cfRNA level due to growth of the tumor mass and/or to increased shedding of tumor material into the blood, whereas a decrease in the cfRNA signal often accompanies a radiographic response.

Aside from the use of total cfRNA as a general marker for tumor response, cfRNA may also provide an opportunity for blood-based testing of a correlative for PD-L1 expression that may be at least as reliable as PD-L1 IHC from tissue. Some therapies are thought to be most effective when PD-L1 expression is high (e.g., pembrolizumab or atezolizumab without chemotherapy)^[28]^, and the blood-based test for PD-L1 provides a non-invasive means to identify those patients. The relative gene expression of PD-L1 was obtained by normalizing its PCR signal expression to that of Β-actin and thus was independent of the total cfRNA level. The data presented suggest that cfRNA may also serve as a marker for response in individuals treated with checkpoint inhibitor [Figures 6 and 7].

We acknowledge that this work has limitations as an exploratory analysis with limited numbers and limited clinical data, and although definitive conclusions cannot yet be drawn, this serves as a justification for more substantial research on cfRNA.

In conclusion, although definitive conclusions are not possible from this exploratory cohort, the results are compelling for the potential use of cfRNA in clinical practice and serve as justification for further analysis. An important aspect to highlight is cfRNA levels were measurable in all patient samples in this study, while 12% of patient samples were “out of range” of the assay and not useful for accurate measurement of cfDNA levels. cfRNA levels were measurable in all of the over 2000 cancer patient samples analyzed in multiple tumor types in our experience. These results suggest the use of cfRNA may be a useful supplement in the monitoring of patient disease states. Monitoring gene expression for PD-L1 by cfRNA appears to correlate with RECIST response in patients being treated with a checkpoint inhibitor. As expected, the PD-L1 cfRNA level that correlates with checkpoint inhibitor treatment is not seen when the treatment is chemotherapy or targeted therapy. Monitoring cfRNA expression of Β-actin more broadly correlates with response to treatment in general. A noteworthy concordance was observed between clinical response and changes in cfRNA levels in lung, breast, and CRC patients, independent of the chemotherapy regimen. Changes in relative *PD-L1 *gene expression correlated specifically with the outcome of immunotherapy (90%). We conclude that changing cfRNA levels may indicate treatment response, and PD-L1 may provide valuable information to monitor response to immunotherapy. There is significant potential for cfRNA to provide clinical utility as a blood-based correlate to PD-L1 for treatment planning and as an ongoing biomarker for response assessment in patients being treated with a checkpoint inhibitor. General cfRNA expression of Β-actin may provide an opportunity for ongoing disease assessment. The greater sensitivity relative to cfDNA in a group of patients in this study also highlights a potential advantage for MRD assessment. Our results suggest that there is considerable potential for cfRNA detection related to cancer care. Further research is needed in all of these settings to help guide where adoption in practice may be warranted.

## DECLARATIONS

### Copyright

© The Author(s) 2021.

## References

[1] Fey MF, Tobler A (1996). Tumour heterogeneity and clonality--an old theme revisited. Ann Oncol.

[2] Rolfo C, Castiglia M, Hong D (2014). Liquid biopsies in lung cancer: the new ambrosia of researchers. Biochim Biophys Acta.

[3] Crowley E, Di Nicolantonio F, Loupakis F, Bardelli A (2013). Liquid biopsy: monitoring cancer-genetics in the blood. Nat Rev Clin Oncol.

[4] Diaz LA Jr, Bardelli A (2014). Liquid biopsies: genotyping circulating tumor DNA. J Clin Oncol.

[5] Umetani N, Kim J, Hiramatsu S (2006). Increased integrity of free circulating DNA in sera of patients with colorectal or periampullary cancer: direct quantitative PCR for ALU repeats. Clin Chem.

[6] Kuang Y, Rogers A, Yeap BY (2009). Noninvasive detection of EGFR T790M in gefitinib or erlotinib resistant non-small cell lung cancer. Clin Cancer Res.

[7] Jian G, Songwen Z, Ling Z (2010). Prediction of epidermal growth factor receptor mutations in the plasma/pleural effusion to efficacy of gefitinib treatment in advanced non-small cell lung cancer. J Cancer Res Clin Oncol.

[8] Taniguchi K, Uchida J, Nishino K (2011). Quantitative detection of EGFR mutations in circulating tumor DNA derived from lung adenocarcinomas. Clin Cancer Res.

[9] Gautschi O, Huegli B, Ziegler A (2007). Origin and prognostic value of circulating KRAS mutations in lung cancer patients. Cancer Lett.

[10] Sawyers CL (2008). The cancer biomarker problem. Nature.

[11] Osumi H, Shinozaki E, Yamaguchi K, Zembutsu H (2019). Early change in circulating tumor DNA as a potential predictor of response to chemotherapy in patients with metastatic colorectal cancer. Sci Rep.

[12] Bettegowda C, Sausen M, Leary RJ (2014). Detection of circulating tumor DNA in early- and late-stage human malignancies. Sci Transl Med.

[13] Herbst RS, Soria JC, Kowanetz M (2014). Predictive correlates of response to the anti-PD-L1 antibody MPDL3280A in cancer patients. Nature.

[14] Hamanishi J, Mandai M, Iwasaki M (2007). Programmed cell death 1 ligand 1 and tumor-infiltrating CD8+ T lymphocytes are prognostic factors of human ovarian cancer. Proc Natl Acad Sci U S A.

[15] Topalian SL, Hodi FS, Brahmer JR (2012). Safety, activity, and immune correlates of anti-PD-1 antibody in cancer. N Engl J Med.

[16] Brahmer JR, Tykodi SS, Chow LQ (2012). Safety and activity of anti-PD-L1 antibody in patients with advanced cancer. N Engl J Med.

[17] Ishiba T, Hoffmann AC, Usher J (2018). Frequencies and expression levels of programmed death ligand 1 (PD-L1) in circulating tumor RNA (ctRNA) in various cancer types. Biochem Biophys Res Commun.

[18] Raez L, Usher J, Sumarriva D (2019). PD2.01 PD-L1 and other potential predictive biomarkers measured in plasma by RT-PCR in cfRNA and cfDNA to monitor clinical responses in metastatic lung cancer patients. J Thorac Oncol.

[19] Wan JCM, Massie C, Garcia-Corbacho J (2017). Liquid biopsies come of age: towards implementation of circulating tumour DNA. Nat Rev Cancer.

[20] Volckmar AL, Sültmann H, Riediger A (2018). A field guide for cancer diagnostics using cell-free DNA: from principles to practice and clinical applications. Genes Chromosomes Cancer.

[21] Hu Y, Ulrich BC, Supplee J (2018). False-positive plasma genotyping due to clonal hematopoiesis. Clin Cancer Res.

[22] Larson MH, Pan W, Kim HJ (2021). A comprehensive characterization of the cell-free transcriptome reveals tissue- and subtype-specific biomarkers for cancer detection. Nat Commun.

[23] Kamm R, Smith A (1972). Ribonuclease activity in human plasma. Clin Biochem.

[24] Kopreski MS, Benko FA, Kwak LW, Gocke CD (1999). Detection of tumor messenger RNA in the serum of patients with malignant melanoma. Clin Cancer Res.

[25] Kopreski MS, Benko FA, Gocke CD (2001). Circulating RNA as a tumor marker: detection of 5T4 mRNA in breast and lung cancer patient serum. Ann N Y Acad Sci.

[26] Das K, Norton SE, Alt JR, Krzyzanowski GD, Williams TL, Fernando MR (2014). Stabilization of cellular RNA in blood during storage at room temperature: a comparison of cell-free RNA BCT(®) with K3EDTA tubes. Mol Diagn Ther.

[27] Abbosh C, Birkbak NJ, Swanton C (2018). Early stage NSCLC - challenges to implementing ctDNA-based screening and MRD detection. Nat Rev Clin Oncol.

[28] Peters S, Dafni U, Perol M (2021). VP2-2021: Effectiveness of PD-(L)1 inhibitors alone or in combination with platinum-doublet chemotherapy in first-line (1L) non-squamous non-small cell lung cancer (Nsq-NSCLC) with high PD-L1 expression using real-world data. Ann Oncol.

